# Two Cases of Lithotomy

**Published:** 1859-08

**Authors:** David Prince

**Affiliations:** Jacksonville, Ill.


THE CHICAGO

### MEDICAL JOURNAL.

VOL. II. AUGUST, 1859. No. 8.

### ORIGINAL COMMUNICATIONS.

ARTICLE I.

### TWO CASES OF LITHOTOMY.

Reported chiefly with reference to the Constitutional Treatment in connexion with Operations and Injuries.

BY DAVID PRINCE, M.D., JACKSONVILLE, ILL.

I have in my mind the lessons of my early medical education, enjoining an extreme antiphlogistic treatment and the least possible nourishment in connexion with operations and injuries, *lest inflammation should run too high.* The lessons of experience have greatly modified those of my early education.

CASE 1.—Marshall Sharpe, aged 21, of rather short stature and solid make, with a bluish skin, as if there were a rather sluggish capillary circulation, and walking carefully, as if afraid of any jar, says that ever since his earliest recollection he has had some trouble in his bladder. He has the usual symptoms of stone, and gives the test by sounding.

*Jan. 25th*, 1857. After five days of spare diet, with a quarter grain each of morphia and belladonna at bedtime, to secure good sleep of nights, and after taking a dose of oil the night before to effectually evacuate the bowels, and having been etherized (with ether $\frac{3}{4}$ and chloroform $\frac{1}{4}$ mixed), Drs. Edgar, Jones, Gaddis and Christie being present,—the patient being confined in the usual position, and the presence of the stone having been demonstrated by the click against the sound—the ordinary lateral operation was proceeded with, using a scalpel to open into the staff, and a long, straight, slender, wide-pointed bistoury to divide the prostatic body. Four stones were extracted, with an aggregate weight of a little less than three ounces (922 grains).

There was nothing remarkable in the operation, and at its close every circumstance seemed favorable. Pulse 76 in the after part of the day. A tolerably comfortable night followed.

*Second day.* Vomited in the morning after drinking considerable water; pulse 90.

At noon, a chill followed by a fever, with a pulse of 120; sweating off by six o'clock, with a soft pulse of 100.

Eats a little toast with relish. This is the first nourishment he says he has taken for two days except gruel, having an idea that he could not starve himself too much.

He says he had a chill two weeks ago, which throws some light upon the present one. 5 grs. quinia to be taken to-night, with 20 drops laudanum, and 5 grs. quinia to be taken in the morning.

*Third day.* After a tolerable night, pulse 100 in the morning and 108 in the evening; eats his toast with only a moderate relish; no pain; some urine passes the natural way. Oil and laudanum to be taken at bedtime.

*Fourth day.* Oil operated; pulse 96; complains of nothing but weariness from lying; takes 5 grs. quinia this morning.

*Fifth day.* Pulse 88.

*Sixth day.* Morning.—Has slept poorly; urine passes almost entirely the natural way; has a troublesome pain in the back. Evening.—Pulse 100. Take oil and laudanum.

*Seventh day.* Slept better. Oil not operating, takes two laxative pills; not operating, the dose was repeated with the desired effect. Pulse 100.

*Ninth day.* Slept well; pulse 100. Urine passes mostly through the wound. Takes 3 grs. quinia in morning.

9 *p. m.* Pulse 120. Tongue assuming a brownish color. Takes quinia 3 grs. and laudanum 20 drops.

*Tenth day.* 9 *a. m.*—Slept well; pulse 120; feels weak. Takes during the day, wine at intervals, some chicken and some beef tea.

9 *p. m.* Pulse 100. Says he feels stronger. His living has agreed with him, though he has found no good taste in his chicken or in his beef tea.

This is significant. Here has been a mistake, growing out of the assumption, that the appetite would indicate the necessity of the system for food. This patient has not taken sufficient food for several days, simply because he was not told that he must eat it *whether he liked it or not.* This want of appetite has probably been aided by the force of imagination. Once get a patient to believe that the less he eats the better it will be for him, and if his case is so serious as to engross his mind, he will probably think he has no relish for food, just as there is no relish for fun, either in a revival, or upon a death-bed.

I wrote in my case book: "He seems for the last three days to have been sinking from inanition, but I have been afraid of stimulants, lest they should excite local inflammation; and he has had no appetite for any food. Finding the urine to cease entirely by the urethra, and to come altogether by the wound, I inferred that repair had ceased for want of the material in the blood with which to effect reparation, and the trial of to-day seems to show that I was carrying the antiphlogistic regimen too far."

He takes as a retiring dose, quinia 3 grs., laudanum 15 drops, and brandy a teaspoonful.

He is to have beef-tea and brandy once during the night.

*Eleventh day.* Pulse 92 in the morning, and 100 in the evening. Slept well, and says he feels better. No appetite; coating remains upon his tongue; urine all passes by the wound.

Takes 3 grs. quinia three times a day; a teaspoonful of brandy and two or three tablespoonfuls of beef tea, about every three hours.

*Twelfth day.* Pulse 80, both morning and night; sits up in a rocking chair for a short time; takes his beef tea freely, and it is *less offensive to him than at first.* Takes also pretty freely of wine and a small quantity of brandy. Two evacuations from his bowels without cathartic medicine.

Though he has not relished anything up to this time, yet nothing has distressed him after it has been taken. I am reminded by this of the experience of Dr. Kane, who spent two winters near the 80th parallel with his party, undergoing great privations. He states that when suffering from inanition from deficient food and hard exercise, the appetite and relish for food became extinct, and he had to require his men to eat *against their inclination.*

Had I persisted in my fears of inflammation, and allowed my patient to starve himself, I should certainly have lost him. The case had come into that typhoid condition, so commonly arising from general causes, in which the success of the physician depends upon his skill in feeding.

From this time my patient gained appetite and strength rapidly, and there was no further any trouble.

CASE 2.—Daniel McLaughlin, aged 26, of moderate stature and spare habit, presented himself six years ago, and on sounding, a stone was distinctly felt. He was advised to be cut, but did not like the advice. He afterwards spent two summers at the mineral springs in Green county, near Whitehall, and found great relief from the water. He thought himself cured, got married, went to farming, and begat children. He could ride on horseback, and do everything that an active and energetic man wishes to do.

Last harvest, while working hard, he got an irritation of his bladder with what was supposed to be an impaction of a stone in the neck of the bladder. After several days, his physician supposed that he pushed away the stone by means of a catheter, and the patient was relieved. He was, however, unable to do any more work, and continued feeble, having frequent attacks of chills, with a necessity to pass his urine from ten to twenty times every night.

As a last resort, he resolved to submit to an operation. For this purpose he presented himself, January 24th, 1859, with a habitual pulse of 100, emaciated, and unable to go up and down stairs without a great aggravation of the vesical irritation.

Having resolved upon the operation, he was in great haste to have it over with; but he was told that his pulse must come down to 70, and that this must require rest and time. By morphia to procure sleep, by non-mercurial laxatives to neutralize the constipating effects of the morphia, by moderately nutritious but easily digested farinaceous diet, I hoped to bring the pulse down to the test frequency of 70. In spite of this, his pulse remained about 90. The urine being acid to test paper, I gave him a decoction of uva ursi, with a small quantity of carbonate of soda. Not being satisfied with my treatment in quieting irritation, I gave him three grains of quinia three times a day, with a manifest influence in reducing the frequency of the pulse. The first day's trial working well, he took fifteen grains on the second day, but his pulse would not come below 80.

*Feb. 2nd.* Patient has less irritation, and is anxious for the operation. I had the night before resolved to operate if the wind would change from the warm, damp, negatively electrified south wind, to the cold, dry, positively electrified north-west. On rising in the morning, the wind was still blowing from the south; but by 7 o'clock it had changed to the desired north-west.

I have a suspicion that in operations there is not sufficient attention paid to such considerations; but when the question of success or failure turns upon a combination of influences, each one slight in itself, attention to them will often give the slow and clumsy operator far more reputation than manual dexterity can secure. In operations which can be done one day as well as another, it may be of no little importance to choose a day in which the warmly dressed man will say, "how fine and bracing," instead of a day in which he will say, "how dull and good-for-nothing it makes one feel."

The pulse was still at 80, ten beats above my test number, but knowing that it was kept up by an irritation which the operation would in part remove, I proceeded. Present: Drs. Long, Reed, English and G. Selden Smith, and some non-professional gentlemen. Patient etherized in bed to save the repulsion of being laid upon the table in the waking state (ether $\frac{3}{4}$, chloroform $\frac{1}{4}$, mixed). Operation proceeded with in all respects as in the other case just detailed, only that warm water to the amount of a pint was thrown into the bladder previous to cutting. The application of the forceps revealed a stone larger than had been estimated. Forceps loosened from the stone and withdrawn. Bistoury reintroduced, guided by the forefinger of the left hand, and made to cut the prostatic body upon the right side, taking great care not to go beyond the prostatic investing membrane. This was done without any further enlarging of the external wound, and after the manner described by Dr. R. D. Mussey, in the *American Journal of Medical Sciences*, for January, 1844, page 264, and by Dr. J. C. Warren, in the same journal, for October, 1844, page 293. A larger forceps was introduced, and aided by the forefinger of the left hand in the rectum, and much patient waiting and gradual pulling, after the fashion of traction upon the child's head with obstetrical forceps, the stone was finally brought out, of a brownish color, as of lithic acid, measuring over three circumferences, —6, 7 and 8 inches, and weighing four and a quarter ounces avoirdupois.

The patient was about an hour and a half under the influence of ether, and after being put to bed his pulse was 150 and feeble; but giving several tablespoonfuls of whisky, and covering him up well with bed clothes, the pulse gained in force and fullness and lessened in frequency.

10 *p. m.* Pulse 100; pain in abdomen, but no marked pain on pressure. He has taken some brandy and broth during the evening. I introduced a portion of gum elastic catheter to ensure a drain from the bladder. Takes morphia $\frac{1}{4}$ grain in a tablespoonful of wine.

*Second day.* 5 *a. m.*—Pulse 100, small; has rested pretty well during the night; urine passes partly through the catheter and partly outside of it. He has taken a little wine several times, and ate a piece of toast with a cup of coffee. Says he has not as good a relish as yesterday morning, before the operation.

3 *p. m.* Pulse 100; some thirst. Says he feels faint and dizzy at times. Does not drink as much to-day as yesterday.

*Third day.* 7 *a. m.*—Pulse 100, soft. Has just taken a cup of coffee and ate a piece of toast. Skin of natural feel; some hoarseness in voice.

1 *p. m.* Pulse 100. Has taken more nourishment to-day than yesterday. He has taken tincture of veratrum viride, at 7 a.m. six drops, at 9 six drops, and at 1 p.m. twelve drops, making in all twenty-four drops. At the time of giving this last dose there was no apparent effect from the others.

5 *p. m.* Pulse 56; vomiting greenish fluid; complains of being deathly sick. He is overdosed with veratrum viride. Wine and morphia first given were thrown up, but half a grain of morphia given at 7 o'clock remained, producing a comfortable sleep.

8 *p. m.* Pulse 56.

*Fourth day.* 7 *a. m.*—Pulse 88. Has passed a good night. Eats his toast with a good relish.

11 *a. m.* Carefully changed from one bed to another.

9 *p. m.* Pulse 80; cheerful; eats his food with relish. Feels an occasional discharge of wind through the wound, indicating a fistula made either by cutting or tearing.

*Fifth day.* Pulse 80; good appetite; eats some meat.

*Seventh day.* Pulse 88. Six grains of leptandrin given last night have not operated, and he takes oil which operates well.

*Eighth day.* Pulse 80.

*Ninth day.* 7 *a. m.*—The wind has been blowing briskly from the north-west, bringing the temperature from 7° yesterday to zero to-day. Patient had a chill in the night, which woke him out of his sleep, leaving him with tenderness of the abdomen. No appetite; pulse 92. Gave one grain of morphia with hot applications to his abdomen, followed soon afterwards by a dose of sulphate of magnesia.

2 *p. m.* Pulse 112; sweating. He has been thirsty, but is now less so.

4 *p. m.* Pulse 100. Sulph. magnesia has operated. Severe tympanitis of the abdomen, and tongue and lips somewhat dry.

11 *p. m.* Pulse 108. Has taken three drops tinct. veratrum viride at 7 and 10, and now takes three drops veratrum viride, seven grains of quinine, and one grain of morphia, all in one dose.

12 *Nocte.* Skin of healthy softness. Takes three drops tinct. veratrum viride and eats a little.

*Tenth day.* 7 *a. m.*—Pulse 100. Alvine evacuations thin, and passing with but very little consciousness on the part of the patient. Ordinary respiration good, but a deep breathing is painful; pain felt in hypogastric region; tympanitis and soreness less. Takes three drops veratrum viride in a little wine.

9 *a. m.* Takes six grains quinia, one quarter grain morphia, and three drops veratrim viride, in wine; shortly afterwards, five grains acetate of lead in wine; soon afterwards, a tablespoonful of brandy, with very manifestly good effect both upon the feelings of the patient and in restraining the diarrhœa.

5 *p. m.* He has taken some toast and tea, and has slept well. Vomited about 12 o'clock, after taking some brandy, but he immediately took more, which remained upon his stomach.

10 *p. m.* Pulse 100. Has taken some nourishment and slept some. Takes one-third grain morphia in some brandy.

*Eleventh day.* 6 *a. m.*—Pulse 98, soft. Has taken considerable nourishment in small quantities. His extremities carefully bathed in warm water.

10 *p. m.* Pulse 98. Nourishment and brandy have been frequently repeated. Had two thin alvine evacuations in the middle of the day. Took three grains quinia and one quarter grain morphia some time during the day. Now eats a little toast. His nourishment is *urged upon him;* he has not much relish for it.

*Twelfth day. Feb. 13th.*—Temperature 3° below 0. Pulse remains about 100. Takes nourishment and is comfortable. About 6 p. m. he had a chill, followed by a fever of about two hours' duration. After this he took one-third grain morphia with three drops veratrum viride, and, not sleeping, the dose was repeated about 10 o'clock. Blister applied to the hypogastrium.

*Thirteenth day.* The drawing blister kept the patient awake more than usual. Pulse 92 in sleep and 100 awake. Took three doses of morphia during the night, and three grains quinia. Somewhat nauseated; vomited about 10 a.m., after taking some nourishment, and had a slight chill in the evening.

*Fourteenth day.* General appearance improved. Toast, beef tea, brandy and morphia are the remedies. Some improvement in his relish for nourishment; has had several slight chills, followed by febrile flushes, during the day.

*Fifteenth day.* Doing well; appetite good.

*Sixteenth day.* Pulse 72; appetite good. A cathartic given.

*Twenty-first day.* Pulse 76 while sitting up and 60 while lying down.

*Twenty-sixth day.* Urine passes partly by the urethra.

*Thirty-fourth day.* Divided the sphincter ani to cure the fistula made in the operation.

*Thirty-fifth day.* Another chill treated by quinia and morpha.

*Forty-seventh day.* He rides out.

*Fiftieth day.* Gone home.

*One hundredth day.* There is still some leak from a small recto-urethral fistula which remains. He has had repeated chills since he went home.

It will be seen from the history of these cases that the treatment of the inflammation, as commonly understood, was a very small element. The practical end forcing itself upon the attention of the surgeon was to keep the patient from *running down.* The general health was below par from the long continued irritation, and the deranging influence of the operation tended to adynamic fever, requiring particular attention to secure the taking of adequate nourishment with stimulating and tonic medicines. In the preparatory treatment of the second case, quinia brought down the frequency of the pulse, while rest, anodynes and moderate diet failed to do it. After the operation, this patient passed no day without taking wine or brandy; and much of the time quinia, with food *against his taste*, and not because he liked it. In the first case, the turning point in the patient's recovery was when nourishment and stimulants were pushed in spite of his loathing of food, and even occasional vomiting.

The condition of these patients is only an exaggeration of the condition of the majority of persons who become the subjects of injuries in our climate; and the surgeon who would be successful, must keep a constant watch lest his patient fail of adequate nourishment. To the man of experience, it will generally be possible to distinguish the cases which really require antiphlogistic treatment. According to my experience, however, it is safer to *anticipate* sinking from the shock of the injury (or operation), and from the tax upon the system involved in the reparation, than to anticipate high inflammation and destructive synochal fever.

As our idiopathic diseases mostly require a treatment which is very far from being antiphlogistic, so the treatment in connection with injuries and operations requires a like modification from that which may be proper when the diseases are of a high inflammatory character.

While it is imperative that watchful care should be exercised to see that the patient is properly supported and nourished, it is also of the very first importance not to neglect *depurative* means. A mercurial cathartic is often the necessary precursor to the administration of tonics and nourishment, and it seems, to a superficial observer, as though calomel were itself a tonic. The excessive *good eater* leaves his hotel and visits a mineral spring; drinking freely of the diuretic water, which, by its dissolving property, clears out the effete materials, and stimulates the processes of transmorphosis throughout the whole system, and he says, "how clarifying, how bracing, how appetizing!" Proper diet and exercise might have obviated the necessity for all this, but the medical man finds his patient in a morbid state, and these artificial means cut short the processes which diet and care may more slowly secure. It so happens, in vast numbers of diseases, accidents and operations, that the question of success is a question of time, just as much as the question whether a horse pulls a cart out of the mire turns on this other question, whether there is a shoulder to the wheel *in time*, before the horse is exhausted.

I have often thought that the skill of one physician over another turned more upon the right use of *depurants* and alteratives than upon anything else. In a plain case of exhaustion it is easy to give stimulants, and in an inflammatory fever to bleed; but when to give calomel, squills, potassa and so forth, so as to make them indirect tonics, and at the same time to be confident that one is *right*, this is an excellence of the highest order in medicine.

